# The impact understanding of exosome therapy in COVID-19 and preparations for the future approaches in dealing with infectious diseases and inflammation

**DOI:** 10.1038/s41598-024-56334-5

**Published:** 2024-03-08

**Authors:** Zeynab Nasiri, Hoorieh Soleimanjahi, Nafiseh Baheiraei, Seyed Mahmoud Hashemi, Mahmoud Reza Pourkarim

**Affiliations:** 1 Department of Virology, Faculty of Medical Sciences, Tarbiat Modares University, Tehran, Iran; 2 Department of Anatomical Science, Faculty of Medical Sciences, Tarbiat Modares University, Tehran, Iran; 3 Department of Immunology, School of Medicine, Shahid Beheshti University of Medical Sciences, Tehran, Iran; 4 Laboratory for Clinical and Epidemiological Virology, Department of Microbiology, Immunology and Transplantation, Rega Institute for Medical Research, KU Leuven, 3000 Leuven, Belgium

**Keywords:** Cytokine storm, SARS-CoV-2, Exosome, COVID-19, PBMC, Stem-cell research, Viral infection

## Abstract

Cytokine storms, which result from an abrupt, acute surge in the circulating levels of different pro-inflammatory cytokines, are one of the complications associated with severe acute respiratory syndrome coronavirus 2 (SARS-CoV-2) infection. This study aimed to assess the effect of exosomes on the release of pro-inflammatory cytokines in patients with coronavirus disease 2019 (COVID-19) and compare it with a control group. The cytokines evaluated in this study were TNF-α, IL-6, IL-17, and IFN-γ. The study compared the levels of these pro-inflammatory cytokines in the peripheral blood mononuclear cells (PBMCs) of five COVID-19 patients in the intensive care unit, who were subjected to both inactivated SARS-CoV-2 and exosome therapy, with those of five healthy controls. The cytokine levels were quantified using the ELISA method. The collected data was analyzed in SPSS Version 26.0 and GraphPad Prism Version 9. According to the study findings, when PBMCs were exposed to inactivated SARS-CoV-2, pro-inflammatory cytokines increased in both patients and healthy controls. Notably, the cytokine levels were significantly elevated in the COVID-19 patients compared to the control group *P*-values were < 0.001, 0.001, 0.008, and 0.008 for TNF-α, IL-6, IL-17, and IFN-γ, respectively. Conversely, when both groups were exposed to exosomes, there was a marked reduction in the levels of pro-inflammatory cytokines. This suggests that exosome administration can effectively mitigate the hyperinflammation induced by COVID-19 by suppressing the production of pro-inflammatory cytokines in patients. These findings underscore the potential safety and efficacy of exosomes as a therapeutic strategy for COVID-19.

## Introduction

The severe acute respiratory syndrome coronavirus 2, which originated in Wuhan, China, in late 2019, soon escalated into a global pandemic, posing a significant threat to public health worldwide. This virus causes a type of pneumonia infection known as coronavirus disease 2019, which became a major global health concern^1^. COVID-19 triggers intense immune responses and promotes uncontrolled cytokine release, resulting in cytokine storms in the lungs^2^. Current evidence suggests that lymphopenia (a condition characterized by low levels of lymphocytes in the blood) and increased levels of circulating cytokines are key factors in the progression of the disease or recovery from it^3–6^. As such, strategies aimed at controlling cytokine release and implementing anti-inflammatory therapy could potentially serve as effective approaches for treating or mitigating pneumonia in COVID-19 patients^7^.

According to the World Health Organization (WHO), the majority of COVID-19 patients exhibit mild (40%) to moderate (40%) symptoms. Conversely, about 15% of patients experience severe symptoms that necessitate oxygen therapy, and 5% develop extremely severe symptoms. These severe symptoms are characterized by serious complications, such as acute respiratory distress syndrome (ARDS), respiratory failure, sepsis, septic shock, and multiorgan failure, which can include cardiac and critical kidney injuries. ARDS is primarily caused by the host’s antiviral inflammatory responses, often triggered by cytokine storm syndrome. This can ultimately lead to multiorgan failure or even death in severe COVID-19 cases^8,9^. Cytokine storms, which significantly contribute to the progression of COVID-19 infection, are sustained and intensified by several concurrent processes, including the activation of antigen-presenting cells (e.g., macrophages), alerting lymphocytes to the presence of the virus, replication of viral RNA within host cells, production of pro-inflammatory factors, and invasion of lymphocytes by the virus, which triggers lymphocyte apoptosis and thus facilitates continuous immune evasion^10^.

Clinical data suggests that cytokine storms induced by COVID-19 involve a self-perpetuating cycle of inflammatory responses, characterized by the continuous release of pro-inflammatory cytokines, such as interleukin-1 (IL-1), interleukin-6 (IL-6), interleukin-12 (IL-12), interferon gamma (IFN-γ), and tumor necrosis factor-alpha (TNF-α), which adversely impact lung tissues^11–13^. Stem cell-based therapies have been recently recognized as innovative treatment strategies for managing patients with COVID-19^14^. A study by Leng et al. was the first to showcase the therapeutic potential of transplanted mesenchymal stem cells (MSCs) in enhancing the pulmonary function of COVID-19 patients^15^. Over the past few decades, the potential mechanisms of MSCs in treating various stages of respiratory diseases have been elucidated^16^. For instance, in multiple ARDS models, the anti-inflammatory and anti-apoptotic properties of MSCs have been shown to improve lung function by restoring epithelial and endothelial cells and facilitating the clearance of alveolar edema fluid. Generally, MSCs are known for their ability to secrete anti-inflammatory cytokines, such as IL-10 and transforming growth factor-β (TGF-β), leading to a reduction in the recruitment of neutrophils into damaged organs and a decrease in the levels of pro-inflammatory cytokines, such as TNF-α, IL-8, and IL-6^17^.

Human umbilical cord-derived (hUC-MSCs) are the primary source of MSCs. According to previous studies, MSC therapies can effectively treat ARDS^18^ and sepsis^19^, which are often severe pathological symptoms in COVID-19 patients. Patients may experience symptoms, such as edema, intrapulmonary shunting, and hypoxemia. However, by regulating the inflammatory activity and preventing apoptosis, MSCs can help halt the progression of the disease^20^. A study by Johnson et al.^21^ demonstrated that human MSC therapy can ameliorate ARDS and sepsis in rats and mice. Furthermore, according to a phase I trial conducted by Zheng et al.^22^ and Wilson et al.^23^, the administration of MSCs did not lead to any adverse side effects. Guo et al. discovered that treating patients with severe COVID-19 symptoms using hUC-MSCs can enhance clinical outcomes by increasing the oxygenation levels and mitigating the incidents of cytokine storms^24^.

Similarly, Liang et al. reported that the allogeneic use of hUC-MSCs can alleviate inflammatory symptoms in patients with COVID-19 pneumonia^25^. Additionally, in their respective clinical studies, both Lanzoni et al.^26^ and Feng et al.^27^ reported encouraging results in COVID-19 patients treated with hUC-MSCs. MSCs utilize three primary mechanisms to manage the symptoms of COVID-19, including immunomodulatory effects, reparative and recovery effects, and antimicrobial effects, particularly in cases of ARDS and sepsis^28^. Despite significant progress, stem cell-based therapies face several obstacles that limit their clinical application, such as immunogenicity, restricted sources of derivation, and ethical considerations. Furthermore, it is widely recognized that the success and effectiveness of stem cell-based therapies in treating COVID-19 largely depend on their paracrine effects and their ability to regulate cytokine storms^14,29,30^.

Exosomes, which are secreted by MSCs, are among the most crucial paracrine effectors. They carry biological cargos similar to their parent cells and possess healing properties, making them appealing substitutes for MSCs in treating various diseases^31^. Exosomes offer several benefits over their parent cells, including their small size, non-toxic nature, low immunogenicity (lacking MHC class I–II), high stability, and ease of storage. Furthermore, exosomes can be easily engineered and manipulated, and they can be produced as readily available products^10,32^. These advantages have contributed to the growing use of exosomes in clinical applications as new therapeutic alternatives^33^.

Exosomes, which are produced by cells infected with SARS-CoV-2, contain viral RNA, key viral proteins, and host cell proteins, such as ACE-2. These components are essential for the virus to enter cells and propagate the infection. This unique characteristic enables exosomes to stimulate both innate and adaptive immune responses, making them potential candidates for vaccines. Additionally, exosomes could serve as effective drug carriers in the treatment of COVID-19^34^. Moreover, exosomes increase the count of anti-inflammatory signaling mediators, which could potentially reduce lung damage by enhancing the functional characteristics and permeability of the alveolar epithelium^35,36^. As a result, the exchange of oxygen-rich air is significantly improved. In addition to their effects observed in preclinical models of acute lung disorders, exosomes derived from MSCs were found to directly inhibit viral replication^35,37^.

Alipoor et al. provided compelling experimental evidence that exosomes, derived from stem cells, have the ability to inhibit signaling pathways associated with hypoxia^38^. This could potentially help reduce inflammation and hypertension as symptoms that are particularly prominent in respiratory diseases. Studies have demonstrated that exosomes, derived from MSCs, produce miRNA. This miRNA acts as a silencing complex and alters the expression of cellular receptors through epigenetic modifications. This process inhibits various RNA viruses, including coronavirus, influenza, and hepatitis C, from invading the body^35,39^. The present study aimed to explore the use of exosomes derived from hUC-MSCs to decrease inflammation in COVID-19 patients in a laboratory setting. This approach has potential applications for other infectious and inflammatory diseases.

## Results

### hUC‑MSC culture

**Figure 1:**
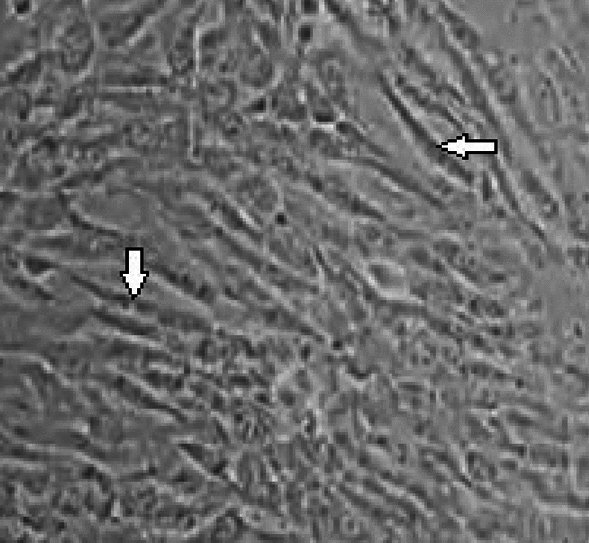
hUC‑MSC morphology in culture conditions (× 100). hUC‑MSC, Human umbilical cord-derived mesenchymal stem cells.

**Figure 2:**
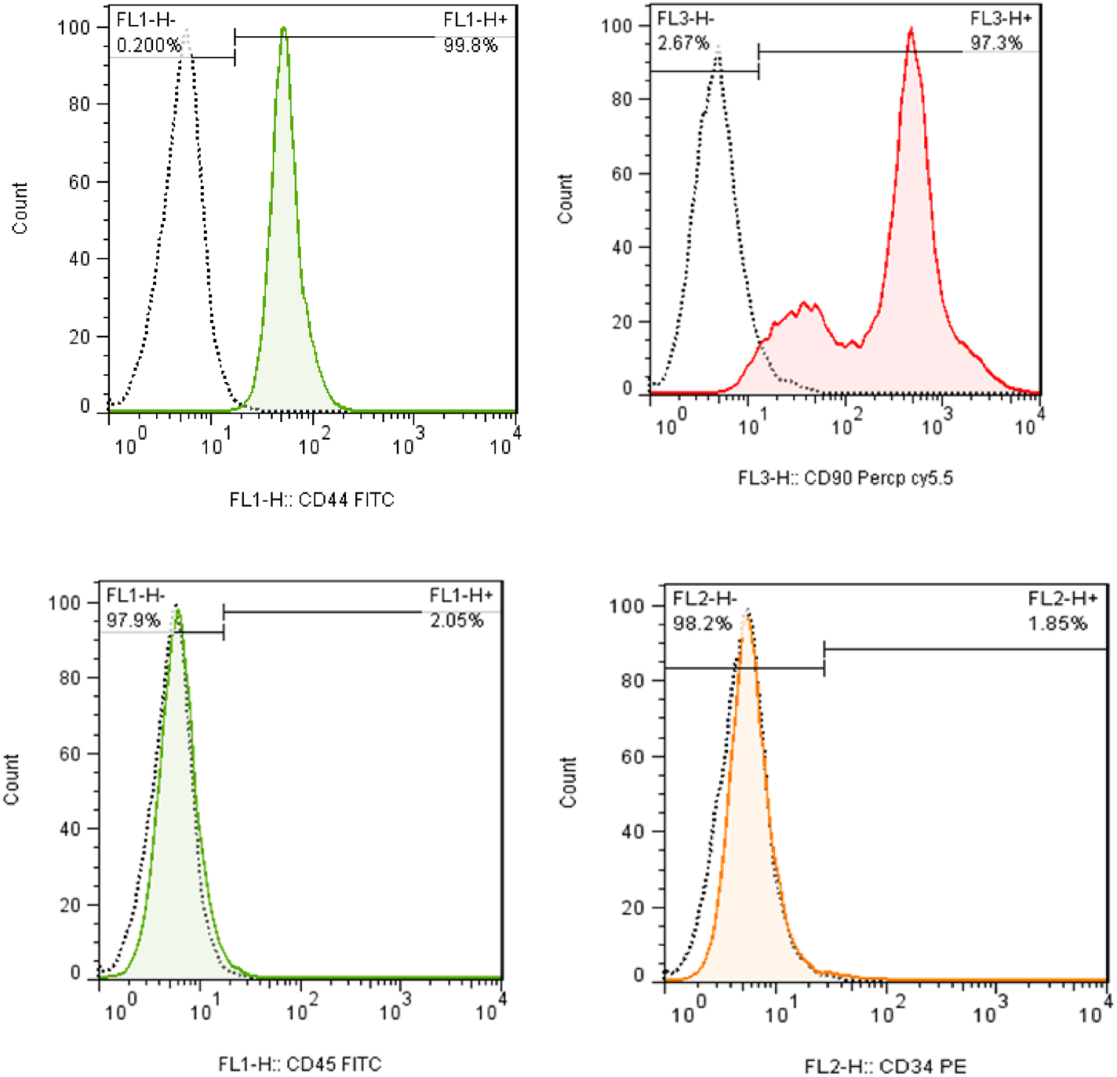
Histogram of a flow cytometric analysis: hUC-MSCs were positive for CD44 and CD90 with percentages of 99.8% and 97.3% of total cells, respectively, and negative for CD34 and CD45 with percentages of 1.85% and 2.05% of total cells, respectively.

The morphology of hUC-MSCs remained consistent when cultured in fetal bovine serum (FBS)-free conditions for exosome generation (Fig. 1). As observed under an inverted microscope, hUC‑MSCs are elongated and spindle-shaped, forming compact clusters of uniform size. We investigated the of the immunophenotypes of MSCs using flow cytometry, a crucial biochemical method for understanding cell types. As depicted in Fig. 2, very high expression levels of CD44 and CD90 were detected, while very low expression levels were observed for CD34 and CD45. These findings align with the criteria set by the International Society for Cellular Therapy for the definition of MSCs.

### Quantification and characterization of exosomes released from hUC‑MSCs

#### Electron microscopy

**Figure 3:**
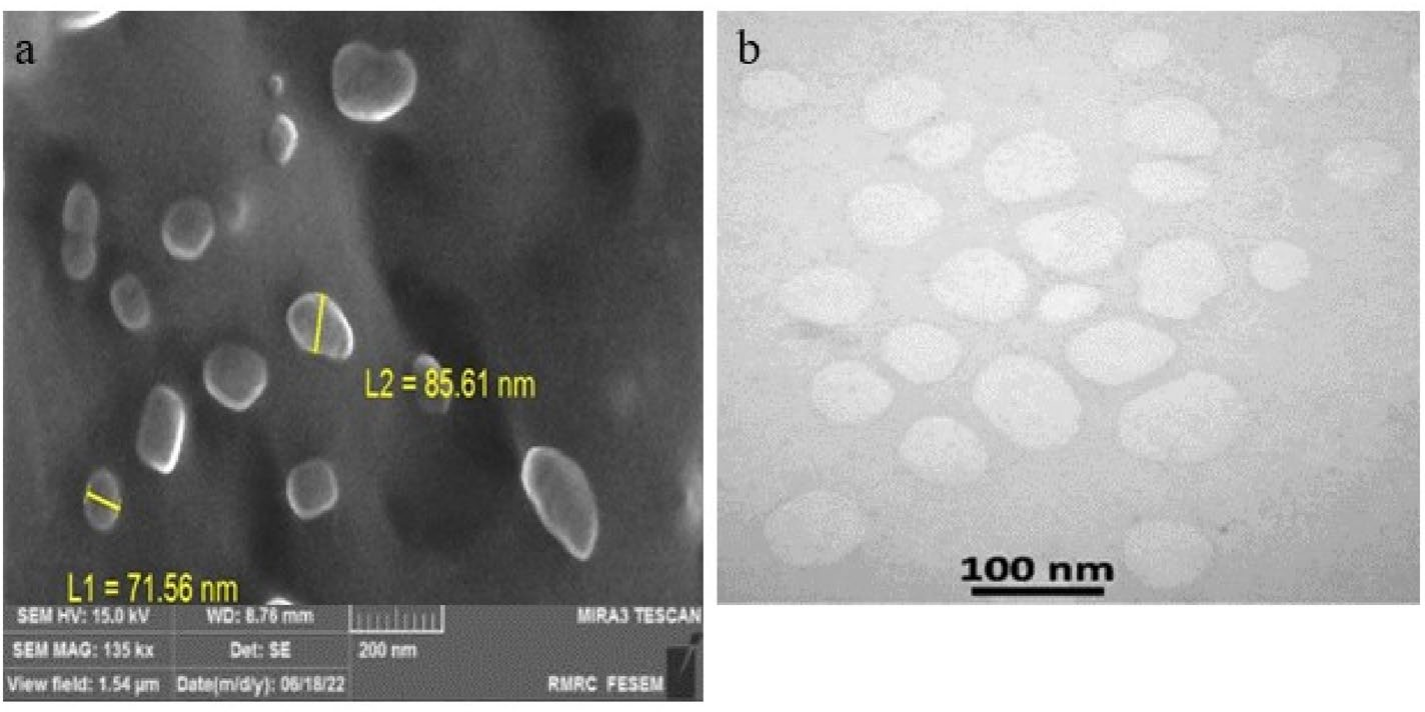
(**a**) FE-SEM images of the isolated exosomes. (**b**) TEM micrographs of isolated exosomes. FE-SEM, Field emission scanning electron microscopy. TEM, Transmission electron microscopy.

Images obtained through field emission scanning electron microscopy (FESEM) confirmed that the exosomes derived from MSCs were small, spherical, and less than 100 nm in size. The morphology of these exosomes was further analyzed using transmission electron microscopy (TEM) with negative staining. The TEM images offered a more intricate view of the exosomes derived from MSCs. These images revealed that the particle pellets were vesicles resembling a cup shape, with membranes attached (Fig. 3a,b).

#### The bicinchoninic acid assay protein assay (BCA)

**Figure 4:**
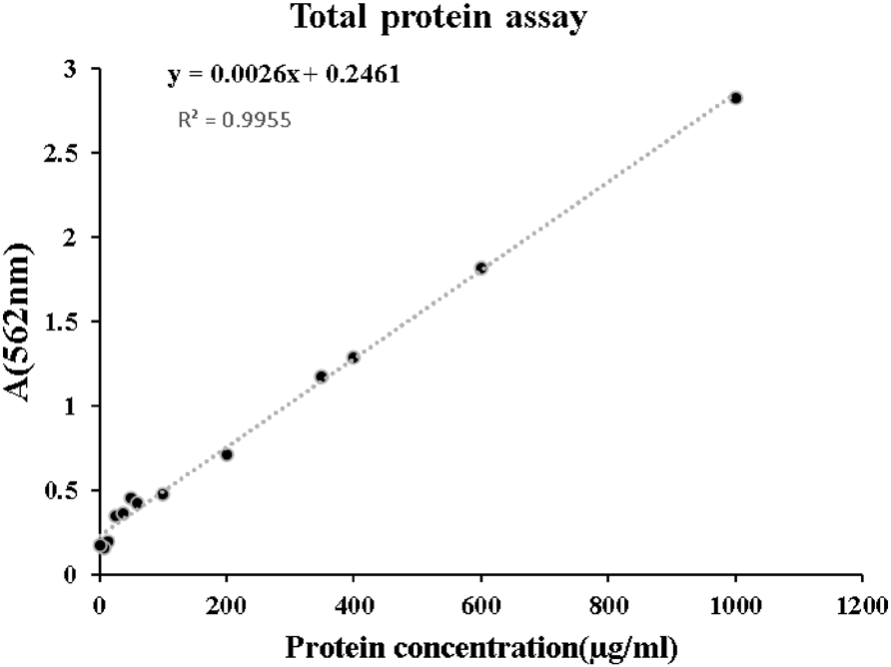
The BSA calibration curve was determined by utilizing the Protein Assay BCA Kit and analyzing the protein concentration. BSA, Bovine serum albumin; BCA, The bicinchoninic acid assay protein assay.

Additionally, the protein concentration was quantified using the BCA method. The resulting value, determined from the protein concentration standard curve depicted in Fig. 4, was measured to be 3482 µg/mL.

#### Dynamic light scattering technology (DLS)

**Figure 5:**
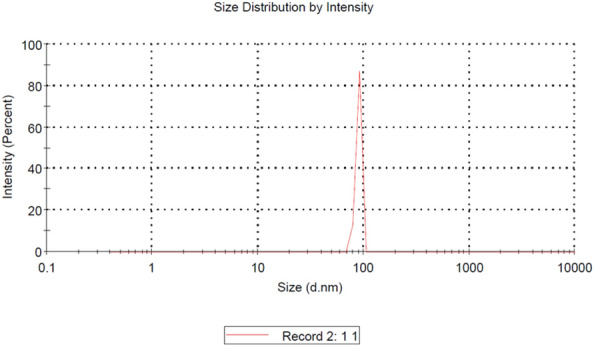
Results of DLS analysis for exosome size distribution. DLS, Dynamic light scattering technology.

The size distribution of the isolated exosomes was determined using the DLS. The DLS analysis revealed that the average size of the exosomes was 89.65 nm, with measurements taken at a constant temperature of 25 °C (Fig. 5).

#### Western blot analysis

**Figure 6:**
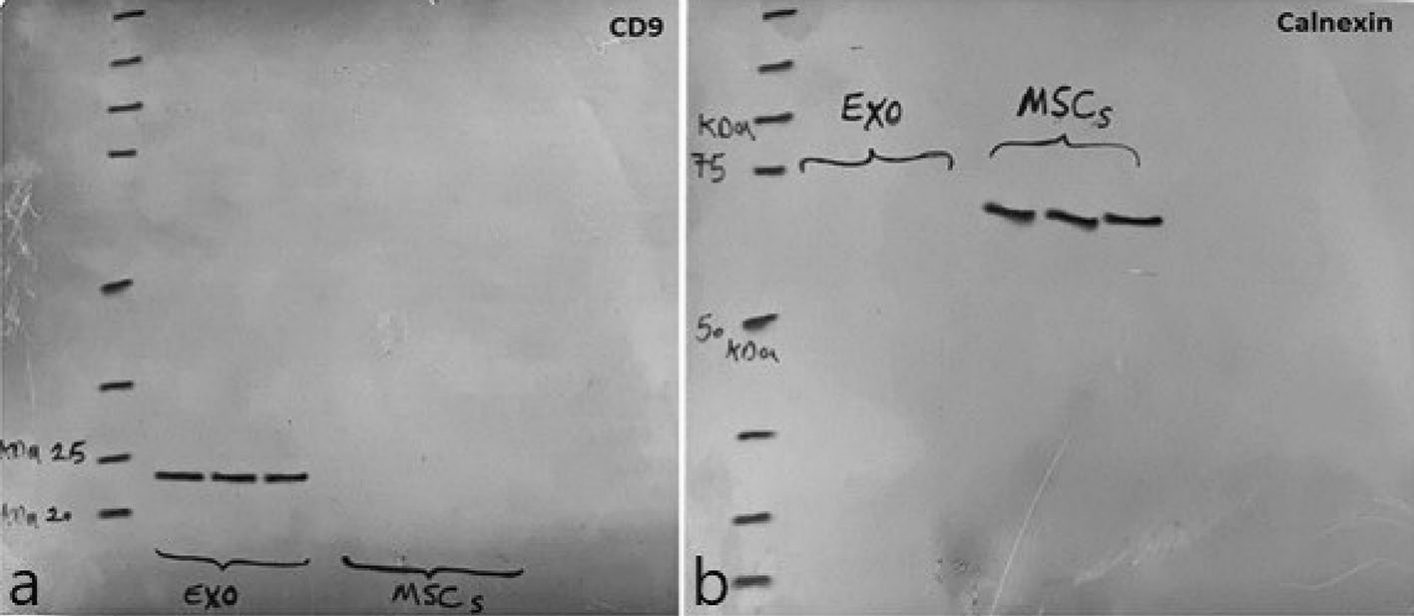
Western blot analysis: (**a**) the presence of the surface marker CD9 (Molecular weight: 24 kDa) in exosome and MSC cells as negative control. (**b**) the Western blot analysis for Calnexin (Molecular weight: 69 kDa) as a negative control in exosomes and positive in MSC cells.

The Western blot analysis detected the presence of CD9, a surface marker typically found on exosomes derived from MSCs. The protein content of the exosomes can be assessed by flow cytometry and Western blot, and the combination of these two methods results in an investigation of both the membrane-bound (CD9, CD63, and CD81) and internalized proteins (Tsg101 and Alix) of the exosomes. Detection of proteins enriched in exosomes, such as CD9, Tsg101, and Alix, and the absence of proteins, such as the endoplasmic reticulum protein calnexin, is an indication that the exosome-enriched pellet is indeed exosomes and not contaminating vesicles from other compartments of the cell as is presented. The exosomes harvested from hUC-MSCs are devoid of any cellular components, as evidenced by the absence of the endoplasmic reticulum chaperone protein, calnexin (Fig. 6).

#### Determination of the dose

**Figure 7:**
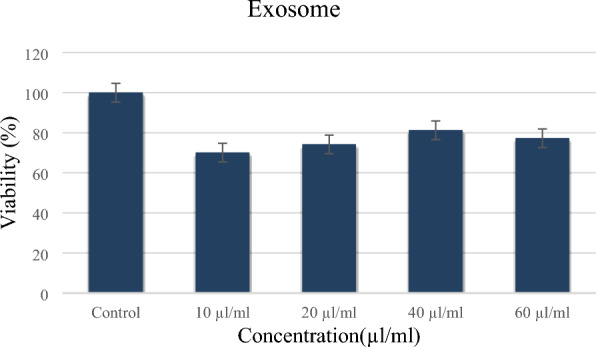
MTT assay of PBMCs incubated with exosomes. Data are presented as the mean viability (%) of triplicate wells with error bars representing the standard deviation (SD). The cell viabilities mean values after 72 h for 10 µL, 20 µL, 40 µL, 60 µL dose of the isolated exosomes and control were 70.04 ± 0.030, 74.14 ± 0.043, 81.23 ± 0.033, 77.24 ± 0.029 and 100.00 ± 0.033, respectively. PBMC, Peripheral blood mononuclear cells.

The assay was employed to identify the optimal dose of the isolated exosomes after 72 h (Fig. 7). Various doses of 10 µg/ml, 20 µg/ml, 40 µg/ml, and 60 µg/ml were evaluated for this purpose. Despite the differences in the calculated viability of the samples not being statistically significant, a dose of 40 µg/mL (the highest dose) was selected as the optimal dose for subsequent experiments.

### Statistical society

Five patients were admitted to the hospital’s intensive care unit (ICU), all of whom tested positive for COVID-19 via the PCR test, with cycle threshold (CT) values ranging between 15 and 25. The healthy controls consisted of five individuals who neither contracted the virus during the epidemic, nor received a vaccine for prevention. In the patient group, three individuals (60%) were men, and two (40%) were women. The healthy group also comprised five individuals, including one (20%) man and four (80%) women.

### Investigation of hematology, biochemistry, and coagulation tests in people with COVID-19

Upon admission to the ICU, the patients were subjected to laboratory tests to determine their white blood cell (WBC) and platelet (PLT) counts, as well as their blood erythrocyte sedimentation rate (ESR), C-reactive protein (CRP), and D-dimer levels (Table 1).

**Table 1:** The results of the tests conducted on COVID-19 patients.

Laboratory test	Unit	Reference interval	Mean ± SD
WBC count	(10^3^/µL)	4.4–11.3	15.34 ± 3.71
Neutrophil	(10^3^/µL)	2.5–6.0	12.55 ± 5.79
Lymphocyte	(10^3^/µL)	1.0–4.8	0.83 ± 1.39
Platelet	(10^3^/µL)	150–450	231.76 ± 125.69
ESR	1h (mm/hr)	0–15	55.29 ± 15.79
CRP	(mg/L)	Negative (≤ 5.0)	145.54 ± 75.24
D-dimer	(µg/mL)	Normal (≤ 0.6)	1.69 ± 0.93
		Borderline (0.6–1.0)	
		Ab Normal (˃1.0)	

### Efficacy of exosome treatment in reducing pro-inflammatory cytokines

**Figure 8:**
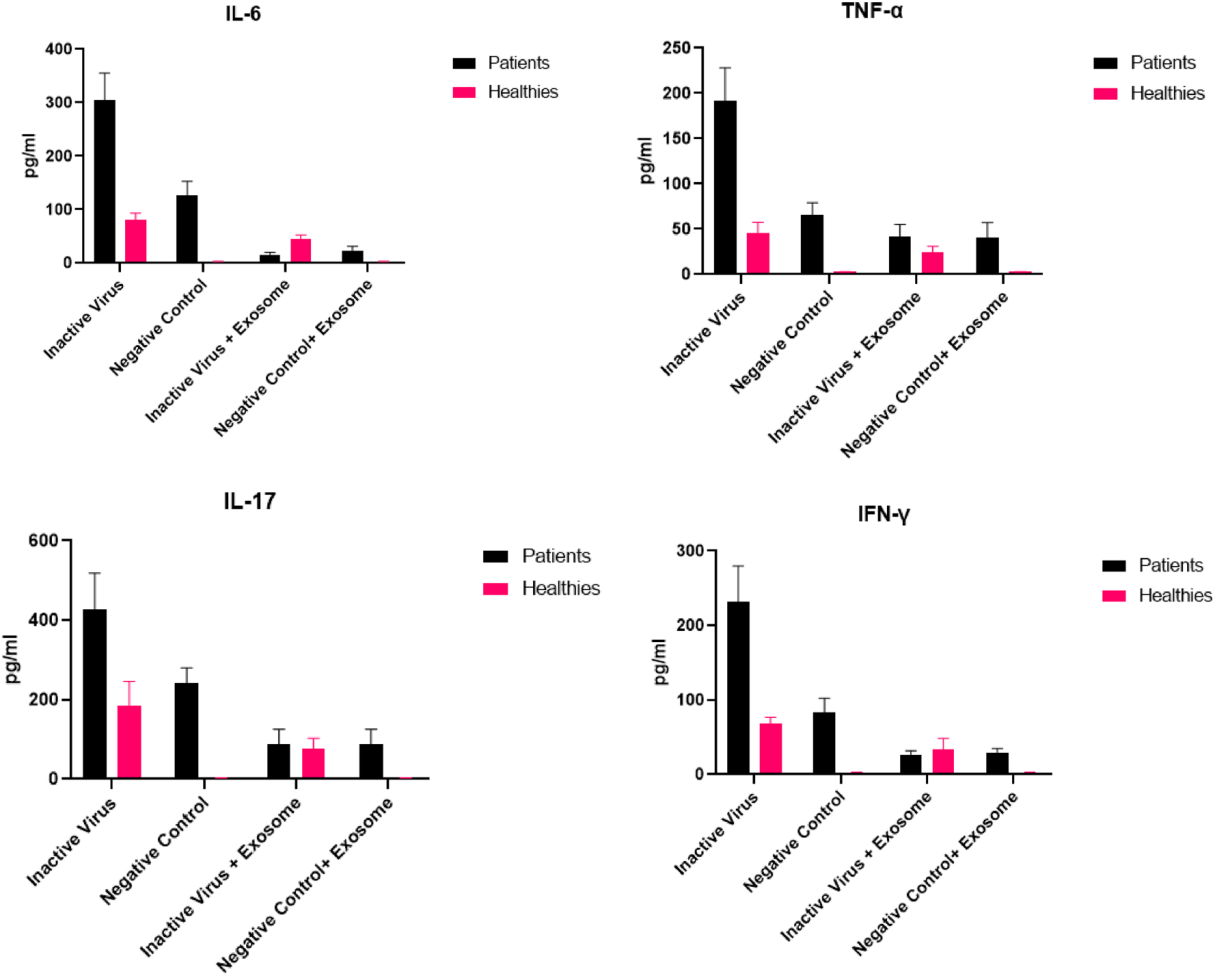
The cytokine expression levels of IL-6, IFN-γ, TNF-α, and IL-17 in COVID‐19 patients and healthy controls. (**a**) Levels of IL-6 are: Inactivated virus = 330.858 ± 79.716, Negative control = 110.822 ± 26.176, Inactivated Virus + Exosomes = 19.248 ± 2.769, and Negative Control + Exosomes = 24.261 ± 5.910 in patients and 68.461 ± 10.889, 1.447 ± 1.112, 39.521 ± 6.221, 1.353 ± 2.036 in healthy participants respectively. (**b**) Levels of IL-17 are: Inactivated virus = 429.064 ± 97.254, Negative Control = 232.208 ± 38.923, Inactivated Virus + Exosomes = 84.824 ± 35.402 and Negative Control + Exosomes = 72.280 in patients and 178.692 ± 58.256, 0.000 ± 0.000, 75.103 ± 24.454, and 0.000 + 0.000 in healthy participants respectively. (**c**) Levels of TNFα are: Inactivated virus = 191.576 ± 46.768, Negative control = 53.248 ± 11.122, Inactivated Virus + Exosomes = 34.669 ± 9.912, and Negative Control + Exosomes = 33.985 ± 11.928 in patients and 37.745 ± 8.928, 0.000 ± 0.000, 22.936 ± 4.242, and 0.000 ± 0.000 in healthy participants respectively. (**d**) Levels of IFN-γ are: Inactivated virus = 233.478 ± 50.475, Negative control = 81.214 ± 19.067, Inactivated Virus + Exosomes = 25.879 ± 5.982, and Negative Control + Exosomes = 28.705 ± 5.984 in patients and 66.534 ± 8.558, 0.383 ± 0.857, 33.320 ± 14.221, and 1.773 ± 1.357 in healthy participants respectively.

The levels of four cytokines were measured in the culture supernatants of PBMCs. These conditions include 1. exposure of PBMCs to the RPMI 1640 medium as a negative control, 2. exposure of PBMCs to the inactivated virus, 3. treatment with exosomes after exposure to the negative control (RPMI 1640 medium), and 4. treatment with exosomes after exposure to the inactivated virus in both patients and healthy participants. To begin with, due to our small sample size, the Shapiro–Wilk test was used to assess the distribution of cytokines' levels in each group in patients and healthy participants. An independent T-test was performed for variables with normal distribution, and for others Mann–Whitney was used to compare the cytokine levels. Results are shown in Tables 2 and 3. All our results demonstrated a significant difference between patient and healthy participants for each condition except for TNF-α, IFN-γ and IL-17 levels in PBMCs exposed to inactivated virus and treated by exosome *P*-values were 0.055, 0.327, and 0.627 respectively. We used paired T-test and Wilcoxon Signed Ranks Test to compare cytokines’ levels before and after PBMCs exposure to the inactivated virus and before and after PBMCs treatment with exosome. We observed a notable decrease in the levels of cytokines among the patient group. Additionally, we found a significant reduction in cytokine levels among healthy controls whose PBMCs were stimulated with inactivated virus. *P*-values and effect sizes for these observations are available in Tables 4 and 5. The findings suggested that when PBMCs were stimulated with the inactivated virus for 24 h, there was a significant increase in the release of pro-inflammatory cytokines, such as IL-6, IFN-γ, TNF-α, and IL-17. Infection with SARS-CoV-2 resulted in the production of pro-inflammatory cytokines. Individuals with symptomatic COVID-19 had higher levels of four cytokines (IL-6, IFN-γ, TNF-α, and IL17) than healthy controls. In Fig. 8, the expression levels of cytokines (IL-6, IFN-γ, TNF-α, and IL17) were displayed for patients and healthy individuals under four different conditions mentioned above.

**Table 2:** Comparing cytokine levels after interventions between patients and healthy controls using independent t-test.

Cytokine	Intervention	Mean difference (SD)	95% Confidence interval	*P*-value
IL-6	Negative control	− 109.375 (11.717)	[− 141.860, − 76.891]	0.001
	Inactivated virus	Nonparametric		
	Negative control + exosomes	Nonparametric		
	Inactivated virus + exosomes	20.273 (3.045)	[12.663 ,27.883]	0.001
IFN-γ	Negative Control	Nonparametric		
	Inactivated Virus	− 166.944 (22.896)	[− 229.173, − 104.715]	0.002
	Negative control + exosomes	− 26.932 (2.744)	[− 33.260, − 20.604]	< 0.001
	Inactivated Virus + exosomes	7.441 (6.900)	[− 9.930,24.813]	0.327
IL-17	Negative control	Nonparametric		
	Inactivated virus	− 250.372 (50.700)	[− 367.286 − 133.459]	0.001
	Negative control + exosomes	Nonparametric		
	Inactivated virus + exosomes	− 9.722 (19.242)	[− 54.095,34.652]	0.627
TNF-α	Negative control	− 53.248 (4.974)	[− 67.058, − 39.438]	< 0.001
	Inactivated virus	− 153.832 (21.293)	[− 202.934, 104.730]	< 0.001
	Negative control + exosomes	− 33.985 (5.334)	[− 48.796, − 19.174]	0.003
	Inactivated virus + exosomes	− 11.734 (4.822)	[− 23.847, 0.379]	0.055

SD, Standard deviation; α = 0.05, *P*-values < 0.05 are significant, n = 10 (5 participants in each group).

**Table 3:** Comparing cytokine levels after interventions between patients and healthy controls using Mann–Whitney U test.

Cytokine	Intervention	Median ± IQR	*P*-value
IL-6	Inactivated virus	176.337 ± 239.582	0.008
	Negative control + Exosomes	11.204 ± 23.609	0.008
IFN-γ	Negative control	29.690 ± 84.977	0.008
IL-17	Negative control	90.536 ± 239.573	0.008
	Negative control + exosomes	19.667 ± 76.379	0.008

*IQR* Interquartile range, α = 0.05, *P*-values < 0.05 are significant, n = 10 (5 participants in each group).

**Table 4:** Comparing cytokine levels after exosome treatment using paired T-test.

Cytokine		TNFα			
Intervention		Negative control	Negative control + exosomes	Inactivated virus	Inactivated virus + exosomes
Healthy (N = 5)					
MEAN (SD)		0.000 (0.000)	0.000 (0.000)	37.745 (8.928)	22.936 (4.242)
Paired Differences	Mean (SD)			14.809 (5.757)	
	95%CI			[7.661, 21.957]	
	t			5.752	
	*P*-value			0.005	
	Cohen’sd			5.757	
PATIENT (N = 5)					
MEAN (SD)		53.248 (11.122)	33.985 (11.928)	191.576 (46.768)	34.669 (9.912)
Paired differences	Mean (SD)	19.263 (3.799)		156.907 (− 43.801)	
	95%CI	[14.545,23.980]		[102.521,211.293]	
	t	11.336		8.01	
	*P*-value	< 0.001		0.001	
	Cohen’s d	3.799		43.801	

SD, Standard deviation; 95% CI, 95% Confidence interval; t, test statistic, Cohen’s d: effect size (0.2 = small effect, 0.5 = medium, 0.8 = large, and 1.3 = very large), α = 0.05, *P*-values < 0.05 are significant, n = 5.

**Table 5:** Comparing cytokine levels after exosome treatment using Wilcoxon.

Cytokine	IL-6				IFN-γ				IL-17			
Intervention	Negative Control	Negative Control + Exosomes	Inactivated Virus	Inactivated Virus + Exosomes	Negative Control	Negative Control + Exosomes	Inactivated Virus	Inactivated Virus + Exosomes	Negative Control	Negative Control + Exosomes	Inactivated Virus	Inactivated Virus + Exosomes
HEALTHY (N = 5)												
Median ± IQR	2.041 ± 2.083	0.767 ± 2.998	65.038 ± 21.040	35.810 ± 11.506	0.000 ± 0.958	2.224 ± 2.665	66.350 ± 16.433	28.032 ± 26.908	0.000 ± 0.000	0.000 ± 0.000	191.348 ± 103.731	83.990 ± 47.362
Z	0.000^c^		− 2.023^d^		− 2.023^b^		− 2.023^d^		0.000^d^		− 2.023^b^	
*P*-value	1		0.043		0.043		0.043		1		0.043	
r	0		0.64		0.64		0.64		0		0.64	
PATIENT (N = 5)												
Median ± IQR	105.976 ± 48.481	24.059 ± 9.521	297.933 ± 112.825	18.380 ± 4.660	80.715 ± 37.273	28.552 ± 10.446	221.607 ± 96.459	27.200 ± 11.522	233.721 ± 73.502	70.820 ± 60.503	412.712 ± 183.304	89.564 ± 63.960
Z	− 2.023^d^		− 2.023^b^		− 2.023^d^		− 2.023^d^		− 2.023^b^		− 2.023^b^	
*P*-value	0.043		0.043		0.043		0.043		0.043		0.043	
r	0.64		0.64		0.64		0.64		0.64		0.64	

IQR, Interquartile range; ^a^Wilcoxon Signed Ranks Test, ^b^Based on negative ranks, ^c^The sum of negative ranks equals the sum of positive ranks, ^d^Based on positive ranks, Z: z-score, r: effect size it is calculated via Z/√N (r figures are interpreted based on following definition: 0.1 = small effect, 0.3 = medium effect, 0.5 = large effect), α = 0.05, *P*-values < 0.05 are significant, n = 5.

## Discussion

This study aimed to investigate the impact of exosomes on the secretion of pro-inflammatory cytokines. Based on our findings, a therapeutic strategy for SARS-CoV-2 infection could effectively involve immunomodulatory treatment to regulate cytokine responses, in conjunction with antiviral treatment. Current evidence suggests that administering exosomes is beneficial for patients with hyperinflammation induced by COVID-19. This is due to the inhibition of the production of several cytokines, specifically IFN-γ and TNF-α, by activated T lymphocytes. The results revealed that the levels of these cytokines were elevated in the culture supernatants of PBMCs from COVID-19 patients compared to those from healthy controls; this suggests that these cytokines could contribute to the pathogenesis of the disease. Furthermore, increased levels of IFN-γ and TNF-α have been linked to the severity of COVID-19, reinforcing their potential role in disease progression. Several clinical studies have found a correlation between the severity and mortality of COVID-19 and hyperinflammation. This hyperinflammation is marked by increased serum levels of pro-inflammatory cytokines and chemokines. Postmortem analyses have shown that high concentrations of these pro-inflammatory cytokines are associated with the infiltration of cells into organs, such as the kidneys, heart, and lungs^40–42^. Furthermore, Han et al. conducted a study where they observed cytokine storms, characterized by elevated serum levels of TNF-α and IL-6^43^. These storms were suggested to be indicators of the severity of the disease. In a similar vein, a retrospective observational study involving hospitalized COVID-19 patients revealed that if the serum level of IL-6 exceeded 30 pg/mL, it was a predictor of the need for invasive mechanical ventilation^44^. Notably, a comparable pattern of cytokine storms was observed in previous outbreaks, such as MERS-CoV and SARS-CoV. The pharmacological adjustment of cytokine hypersecretion in coronavirus infection calls for more research, given the fatalities associated with multiorgan damage^45–48^. Recent clinical studies have indicated that the application of hUC-MSCs yielded favorable outcomes in patients with COVID-19^15,25,49^.

Bone marrow-derived exosomes are novel, multi-targeted, next-generation biological agents, secreted by bone marrow-MSCs^50^. These exosomes are a complex blend of signaling nanovesicles capable of suppressing the cytokine storm and the host’s antiviral defenses, both of which are key features of COVID-19^51^.

Patients with elevated IL-6 levels appear to derive greater benefits from hUC-MSC infusion. This suggests that a more intense inflammatory environment may stimulate the immunomodulatory response of MSCs^52^. Both preclinical and clinical studies have demonstrated that exosomes can mitigate complications arising from cytokine storms in inflammatory diseases, such as ARDS, asthma, chronic obstructive pulmonary disease, and acute lung injury. They achieve this by diminishing alveolar inflammation and edema, while fostering the regeneration of epithelial tissue^10,53–59^.

This study had certain limitations. First, the number of patients included in the sample was limited because of the strict inclusion criteria during the sampling period. Second, this was an in vitro study, and further appropriate trials need to be conducted to test the efficacy of exosomes. Therefore, additional trials are required to investigate the potential of exosomes in improving COVID-19 outcomes in clinical settings.

## Conclusion

It is known that SARS-CoV-2 triggers an overactive immune response, leading to cytokine storms and respiratory distress syndrome, which are significant contributors to COVID-19 morbidity and mortality. MSC-derived exosomes reduce pro-inflammatory cytokines, which are responsible for cytokine storms and strengthen the host's viral defenses against COVID-19. The ability of exosomes to modulate the immune system and facilitate tissue repair, coupled with their small size and targeted transfer capability, render them a promising treatment alternative for COVID-19.

## Materials and methods

### Ethics approval and consent to participate

All participants in the study had the option to participate voluntarily, and their privacy was highly valued. Prior to entering the survey, all participants provided their informed consent for research participation. The participants were guaranteed that their personal information would be kept confidential and not be disclosed. All methods used in the study were in compliance with relevant guidelines and regulations. All methods were conducted in accordance with the Declaration of Helsinki and relevant guidelines and regulations. All experimental protocols were approved by an institutional and/or licensing committee. All experiments followed the guidelines of the Laboratory Ethical Commission of the Faculty of Medical Sciences, Tarbiat Modares University. After receiving Approval No. IR.TMU.REC. 1400.017, the study complies with the rules and regulations. Ethical standards were strictly adhered to during all phases of this research.

### Cell isolation and culture

In this study, umbilical cords were obtained from the Private Royan Umbilical Cord Blood Bank and transferred to the laboratory in phosphate-buffered saline (PBS, Gibco, Germany), containing 100 mg/mL of penicillin and streptomycin antibiotics (Gibco, Germany) under sterile conditions. After being washed, the cords were sectioned into 5-cm pieces each. The blood vessels, which included one large vein and two smaller arteries, were removed, and the Wharton’s jelly was subsequently extracted. The cells were isolated using the explant method. For this purpose, the Wharton’s jelly was formed into small spheres measuring 3–5 mm. They were then cultured in T-25 flasks filled with Dulbecco's modified eagle medium (DMEM, Gibco, Germany), 20% FBS, and 1% Pen-Strep. The cultures were then placed in a CO_2_ incubator at a temperature of 37 °C. The culture media were renewed every three days. After observing tiny clumps of cells around the pieces on days 5–7, the culture medium was renewed every other day for up to seven days^23^. The passage was iterated three times to achieve a uniform cell population. It was then sent to the laboratory to confirm the presence of CD34, CD44, CD45, and CD90 surface markers. Cell surface marker expression in hUC-MSCs was determined from the third passage using a FACSCalibur flow cytometer (BD Biosciences, USA). The anti-human antibodies used for staining included CD34, CD44, CD45, and CD90 (all from eBioscience).

### Isolation and purification of hUC MSC‐derived exosomes

After reaching 80–90% confluency, MSCs at passage 2 were adapted to serum‐free culture by gradually reducing serum concentrations over two weeks. After 48 h, the cell supernatants were collected and filtered through 0.22 μm filters. Exosomes were then extracted using an extraction kit (Exosib, Iran) with two reagents (A and B), according to the manufacturer's instructions. The culture supernatant was mixed with reagent A at a ratio of 5:1, vortexed for five minutes, and incubated at 4 °C overnight. It was then centrifuged at 3500 rpm for 40 min, and the resulting supernatant was discarded. The exosome sediment was mixed with 100 µL of reagent B and stored at − 80 °C for future studies.

### Exosome confirmation methods

#### Electron microscopy

The morphology and size of the exosomes were evaluated via FESEM (MIRA3 TESCAN) and TEM (Zeiss, EM10C). For FESEM imaging, 1 µg/mL of exosome solution was dried on a glass slide for 24 h and covered with a thin layer of gold. For TEM, exosomes derived from MSCs were fixed in paraformaldehyde and glutaraldehyde. Subsequently, they were loaded on a formvar/carbon-covered mesh and contrasted with 2% uranyl acetate. Multiple fields of view were examined for both FESEM and TEM imaging. Representative images were selected based on their ability to accurately allocate the overall characteristics observed in the various fields of view.

#### The bicinchoninic acid assay protein assay

The total exosome content was extracted for quantification with a Bicinchoninic (BCA) Acid Protein Assay Kit (DNA Biotech, Iran), consisting of a standard solution, copper, and BCA reagents. The standard curve was plotted at different levels (50–250 µg/mL) against bovine serum albumin (BSA) as the standard solution. The exosomes and standard solutions were separately mixed with a mixture of copper and BCA reagents at a ratio of 1:50 and incubated at 60 °C for 60 min. Finally, the absorbance of the samples was read with a spectrophotometer at 562 nm (MPR4 + ; Hyperion, Roeder mark, Germany).

#### DLS technology

The size of the exosomes was determined using DLS with a Zeta device (Malvern Instruments, UK). PBS, with a refractive index of 33.1 and viscosity of 1.08, was used as the solvent. These parameters are essential for the device software to analyze the data. This simple, rapid, and nondestructive method, can measure particles ranging from a few nanometers to micrometers. The exosomes were diluted five times, and the sample was then read and analyzed using the Zetasizer APS.

#### Western blot analysis

The protein production was confirmed using the Western blotting analysis. For this purpose, the samples were centrifuged at 14,000 rpm for 20 min at 4 °C to eliminate the lysate. The protein concentration was measured using the BCA Protein Quantification Kit, following the instructions provided by the manufacturer. Equal volumes of lysate and Laemmli 2X sample buffer were combined, and then, 20 µg of this mixture was boiled for five minutes. Subsequently, it was subjected to SDS-PAGE and transferred to a 0.2-µm membrane (Immun-BlotTM, PVDF). Next, the membranes were blocked with 5% BSA in 0.1% Tween 20 for one hour and incubated with anti-CD9 (Cat. No.: ab223052, Abcam) and anti-calnexin control antibodies (Cat. No.: ab133615, Abcam) to show the purity of the extracted exosome from contaminated cellular components, at room temperature for one hour. The membranes were washed with TBST three times and incubated with goat anti-rabbit IgG (H&L) secondary antibody. They were then incubated with enhanced chemiluminescence immunoassay for 1–2 min.

#### MTT assay

The cytotoxicity of exosomes was evaluated using the colorimetric MTT assay. For this purpose, 100 µL of DMEM culture medium containing 10^6^ PBMCs was added to each well of a 96-well plate. The MTT assay was performed after 72 h by adding 100 μL of MTT solution (5 mg/mL in PBS) into each well. The cells were then incubated for four hours. The MTT solution was removed, and 100 µL of dimethyl sulfoxide (DMSO; Sigma‒Aldrich, USA) was added to each well to dissolve the purple formazan crystals. The cytotoxic activity of exosomes was then evaluated by a standard MTT assay. The optical absorbance at 570 nm was measured using a microplate reader (ELISA reader, ELX808, BioTek). The results were reported as the rate of viability based on the concentration curve. All tests were performed in three iterations. The relative cell viability was calculated as follows: Relative cell viability (%) = (ODs/ODc) × 100.

#### Patients and sample collection

The relative cell viability was calculated as follows: Blood samples were collected from five COVID-19 patients admitted to the ICU, who were selected between July and October 2021 in Tehran, Iran. The inclusion criteria were a positive real-time PCR result with a CT value of 15–25, an age range of 20–40 years, and no history of any underlying diseases. Tests for CBC, CRP, ESR, D-dimer, and IL-6 were conducted to ensure that the patients were in similar conditions in terms of disease severity. The exclusion criteria were any dissatisfaction with sampling on the part of the patients and any deterioration in the patient’s condition.

#### Isolation and culture of PBMCs

Three milliliters of blood, treated with heparin, was collected from the patients and then diluted with an equal volume of PBS. This mixture was then added to 2 mL of Ficoll^®^ (Innotriane, Germany) and centrifuged at 2400 rpm for 20 min. The PBMCs, which appeared as a cloudy layer between the Ficoll^®^ and the diluted blood, were carefully collected using a Pasteur pipette. The collected cells were suspended in 2 mL of PBS and centrifuged at 2000 rpm for 10 min to remove Ficoll^®^; this step was repeated at 1000 rpm to remove platelets.

#### PBMCs exposed to inactivated virus

Following a 3-hour incubation period, the PBMCs from each patient were cultivated in six wells. The titer of SARS-CoV-2 was determined to be 10^7.66^ ID_50_/mL. Three of the PBMC culture wells were exposed to 0.3 µL of inactivated SARS-CoV-2, while the remaining three wells, which contained PBMCs but were not exposed to the virus, served as negative controls (RPMI 1640 medium).

#### PBMCs exposed to exosomes

After 24 h, the cell supernatant was collected, centrifuged, and stored at − 80 °C until further evaluation. Subsequently, 40 µg/mL of exosomes was added to the wells containing the control sample from a healthy individual, as well as to the other wells containing patient samples. They were then incubated for 72 h.

### Measurement of cytokines

Following exposure to the inactivated SARS-CoV-2 virus and exosomes, the supernatant from the cell culture was used to measure the levels of IL-6, IFN-γ, IL-17, and TNF-α. This was accomplished using the ELISA method, with the Human Cytokines Measurement Kit.

### Statistical analysis

The gathered data was analyzed using SPSS Version 26.0 and GraphPad Prism 9. An independent sample t-test was employed to compare the mean values between patient and healthy participants. For data with a nonparametric distribution, the Mann–Whitney U test was used for analysis. A *P*-value of less than 0.05 was considered statistically significant. For data with normal distribution and for non-parametrical data Paired t-tests and Wilcoxon were respectively performed to evaluate effects of exosome treatment in cytokines levels variations. Continuous variables were expressed as either the mean with standard deviation or the median with interquartile range. Categorical variables were represented as frequency percentages.

## Data Availability

The corresponding author will provide supporting data via email upon request by editors or referees. All data generated or analyzed during this study are included in the main body of this article.

## Abbreviations

SARS-CoV-2: Severe acute respiratory syndrome coronavirus 2
COVID-19: Coronavirus disease 2019
IL: Interleukin
IFN-γ: Interferon gamma
TNF-α: Tumor necrosis factor alpha
hUC-MSC: Human umbilical cord mesenchymal stem cell
PBMC: Peripheral blood mononuclear cells
SD: Standard deviations
SE: Standard error
CI: Confidence interval
IQR: Interquartile range
PE: Phycoerythrin
FITC: Fluorescein isothiocyanate
Per CP: Peridinin chlorophyll protein complex
