# Author Correction: The impact understanding of exosome therapy in COVID-19 and preparations for the future approaches in dealing with infectious diseases and inflammation

**DOI:** 10.1038/s41598-024-64159-5

**Published:** 2024-06-10

**Authors:** Zeynab Nasiri, Hoorieh Soleimanjahi, Nafiseh Baheiraei, Seyed Mahmoud Hashemi, Mahmoud Reza Pourkarim

**Affiliations:** 1 Department of Virology, Faculty of Medical Sciences, Tarbiat Modares University, Tehran, Iran; 2 Department of Anatomical Science, Faculty of Medical Sciences, Tarbiat Modares University, Tehran, Iran; 3 Department of Immunology, School of Medicine, Shahid Beheshti University of Medical Sciences, Tehran, Iran; 4 Laboratory for Clinical and Epidemiological Virology, Department of Microbiology, Immunology and Transplantation, Rega Institute for Medical Research, KU Leuven, 3000 Leuven, Belgium

Correction to: *Scientific Reports* 10.1038/s41598-024-56334-5, published online 08 March 2024

In the original version of this Article, a previous version of Figure 6 and an incorrect version of its accompanying legend were typeset.

The legend of Figure 6:

“Western blot analysis revealed the presence of the exosome surface marker CD9 (Molecular weight: 69 kDa), whereas calnexin is absent from exosomes.”

now reads:

“Western blot analysis: (a) the presence of the surface marker CD9 (Molecular weight: 24 kDa) in exosome and MSC cells as negative control. (b) the Western blot analysis for Calnexin (Molecular weight: 69 kDa) as a negative control in exosomes and positive in MSC cells.”

The incorrect version of Figure 6 appears below.

**Figure 6:**
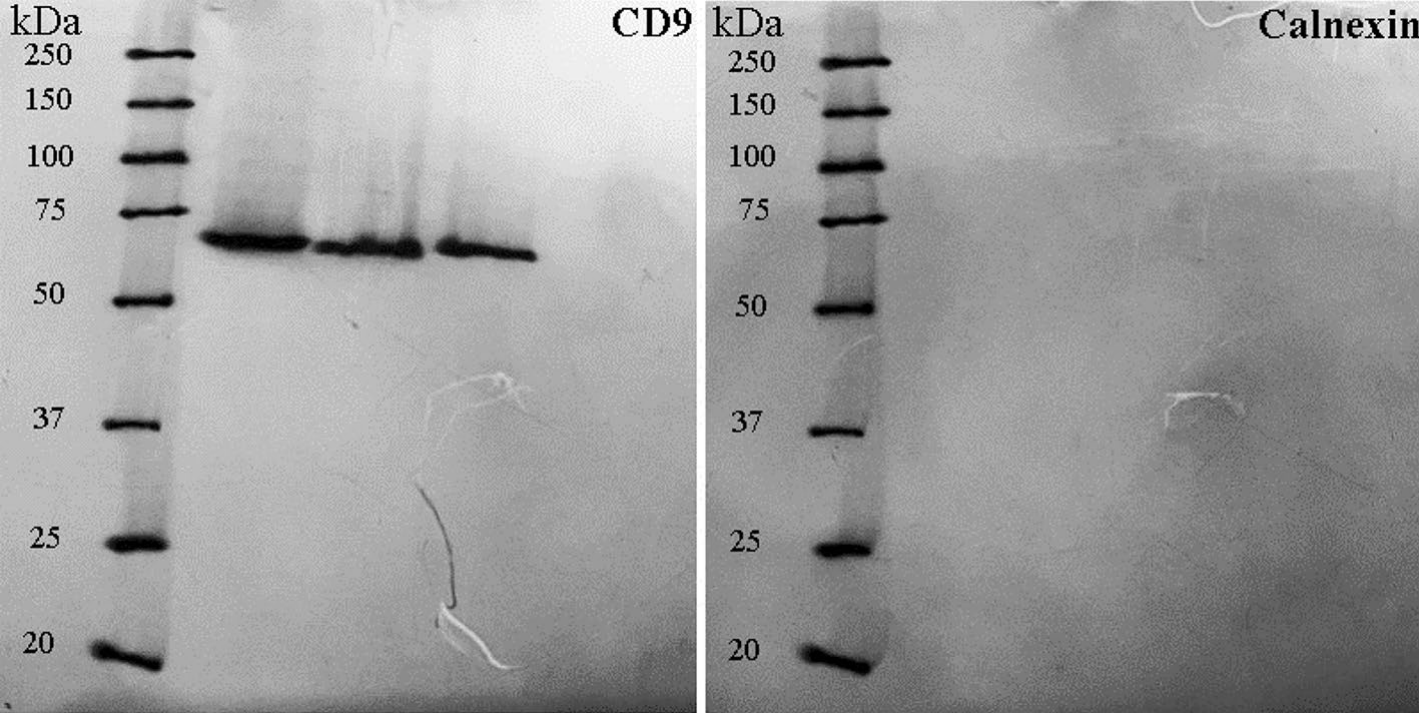
Western blot analysis revealed the presence of the exosome surface marker CD9 (Molecular weight: 69 kDa), whereas calnexin is absent from exosomes.

In addition, in the Materials and Methods section, under the subheading ‘Electron microscopy’, an incorrect Field scanner electron microscopy was referenced.

“The morphology and size of the exosomes were evaluated via FESEM (Hitachi S-4160, Tokyo, Japan) and TEM (Zeiss, EM10C).”

now reads,

“The morphology and size of the exosomes were evaluated via FESEM (MIRA3 TESCAN) and TEM (Zeiss, EM10C).”

Under the subheading ‘Western blot analysis’, the incorrect CD9 was referenced for the Western blotting analysis.

“Next, the membranes were blocked with 5% BSA in 0.1% Tween 20 for one hour and incubated with anti-CD9 (Cat. No.: ab134079, Abcam) and anti-calnexin control antibodies (Cat. No.: ab133615, Abcam) to show the purity of the extracted exosome from contaminated cellular components,”

now reads,

“Next, the membranes were blocked with 5% BSA in 0.1% Tween 20 for one hour and incubated with anti-CD9 (Cat. No.: ab223052, Abcam) and anti-calnexin control antibodies (Cat. No.: ab133615, Abcam) to show the purity of the extracted exosome from contaminated cellular components,”

The original Article has been corrected.

